# P07 Humanizing the transition: improving patient understanding of OPAT and Virtual Wards at Airedale NHS Foundation Trust

**DOI:** 10.1093/jacamr/dlaf239.011

**Published:** 2026-01-05

**Authors:** Fiona Throp, Cheryl Way, Sarah Hall, Kay Green, Sarah Fancy

**Affiliations:** Airedale NHS Foundation Trust, UK; Airedale NHS Foundation Trust, UK; Airedale NHS Foundation Trust, UK; Airedale NHS Foundation Trust, UK; Airedale NHS Foundation Trust, UK

## Abstract

**Background:**

Outpatient parenteral antimicrobial therapy (OPAT) and Virtual Wards can be unfamiliar and anxiety-provoking for patients. Feedback from the 2023 national OPAT conference highlighted the importance of clear, accessible patient information and visible, approachable clinical teams. Patients particularly valued personal connections, reassurance, and opportunities to understand the journey before transfer to community care.

**Methods:**

A focused task and finish group was established, bringing together OPAT clinicians, digital innovation specialists, and patient representatives. Using the Plan–Do–Study–Act (PDSA) cycle, the group designed, tested, and refined a new patient-facing digital resource. Insights from the 2023 OPAT conference and direct patient feedback guided iterative development to ensure accessibility, relevance, and impact.

**Innovation:**

The completed OPAT information webpage provides: (i) four short, animated clips illustrating the patient journey and what to expect on a Virtual Ward; (ii) video introductions from the OPAT team, presenting the real people behind the service and their motivations for working in community care; (iii) a patient story describing lived experience of OPAT, highlighting challenges and benefits; and (iv) QR code access, enabling referring teams to share the resource before discharge or at the point of transition.

**Patient input:**

A patient presentation at the 2023 OPAT conference was central to shaping this work. His ‘top tips’ for OPAT—'sell yourself, provide information on the OPAT team, and include photos’—directly influenced the webpage design.

**Objectives:**

To reduce anxiety during the transition to OPAT/Virtual Ward care; to provide reassurance through clear, accessible information; and to present a welcoming, professional, and approachable community team.

**Conclusions:**

This project shows how a focused task and finish group, supported by the PDSA cycle and digital innovation, can translate patient feedback into practical resources that enhance the OPAT experience. By combining animation, team visibility, and patient voice, the webpage fosters confidence and engagement, supporting a smoother transition from hospital to community care.

**Implications for practice:**

This approach demonstrates the value of integrating patient feedback with digital solutions to improve service communication and offers a scalable model for other Virtual Ward and community healthcare pathways.

